# An explainable multimodal artificial intelligence model for classifying suicide attempters with borderline personality disorder: a pilot study

**DOI:** 10.1038/s41598-025-31550-9

**Published:** 2025-12-19

**Authors:** Claudio Crema, Alberto Boccali, Alessandra Martinelli, Silvia De Francesco, Serena Meloni, Cesare M. Baronio, Roberto Gasparotti, Laura Pedrini, Mariangela Lanfredi, Michela Pievani, Antonino Carcione, Giuseppe Nicolò, Antonino Semerari, Damiano Archetti, Alberto Redolfi, Roberta Rossi

**Affiliations:** 1 Laboratory of Neuroinformatics, IRCCS Istituto Centro San Giovanni di Dio Fatebenefratelli, Brescia, Italy; 2 Unit of Epidemiological Psychiatry and Digital Mental Health, IRCCS Istituto Centro San Giovanni di Dio Fatebenefratelli, Brescia, Italy; 3 Unit of Psychiatry, IRCCS Istituto Centro San Giovanni di Dio Fatebenefratelli, Brescia, Italy; 4 Department of Medical and Surgical Specialties, Neuroradiology Unit, University of Brescia, 25123 Brescia, Italy; 5 Laboratory of Alzheimer’s Neuroimaging and Epidemiology (LANE), IRCCS Istituto Centro San Giovanni di Dio Fatebenefratelli, Brescia, Italy; 6 Department of Human Science, “Guglielmo Marconi” University, Rome, Italy; 7 Third Centre of Cognitive Psychotherapy—Italian School of Cognitive Psychotherapy (SICC), Rome, Italy

**Keywords:** Suicide attempt, Borderline personality disorder, Artificial intelligence, Machine learning, Explainable classifier, MRI, Biomarkers, Diseases, Health care, Psychology, Psychology

## Abstract

Borderline Personality Disorder (BPD) is a severe mental disorder marked by emotional dysregulation. Estimates show that 73% of patients with BPD will have, on average, three suicide attempts in their lifetime, with up to 10% of cases resulting in death. Reliable tools to identify risk factors associated with suicide are lacking. Artificial Intelligence (AI) could fill this gap, supporting the development of effective intervention strategies. This pilot study provides preliminary evidence that a multimodal signature could differentiate suicide attempts in individuals with BPD, paving the way to prospective cohort validation and clinical applications. We developed DRAMA-BPD (Detecting Retrospective suicide Attempts with Machine learning Approaches in Borderline Personality Disorder), an explainable, multimodal, Machine Learning (ML) model based on an ensemble classifier of lifetime suicide attempters among people with BPD. DRAMA-BPD was trained on the sociodemographic, clinical, and MRI data of 104 individuals with BPD recruited from two cohorts. Processing techniques adopted included feature extraction. SHapley Additive exPlanations (SHAP) was used to assess model interpretability. DRAMA-BPD achieved a balanced accuracy of 0.68, sensitivity of 0.58, specificity of 0.77, and AUC of 0.68. SHAP analysis identified cortical volumes and thickness from T1-weighted images and Symptoms Checklist 90 Revised (SCL-90-R) as the main contributors to classification.

**Supplementary Information:**

The online version contains supplementary material available at 10.1038/s41598-025-31550-9.

## Introduction

Borderline Personality Disorder (BPD), a severe mental disorder marked by emotional dysregulation and affect instability, has a prevalence ranging from 0.7% to 2.7% in the general population, with higher occurrence in women^1,2^. It has been estimated that 73% of patients with BPD will have approximately 3 suicide attempts in their lifetime, with up to 10% of cases resulting in death^3–6^. Key risk factors for suicidal behaviors in BPD include impulsivity, self-harm, depressive symptoms, and emotional dysregulation^3,7,8^. While suicide prevention is one of the most important challenges in psychiatric clinical practice, in the field of BPD this aspect is particularly relevant. Indeed, BPD patients often show chronic suicidal ideation that varies in intensity over time, sometimes in association with stressful life events. Suicidal thoughts are very frequent and are not useful in predicting suicidal actions^4^. Given the complex interplay between these factors, identifying those patients who might be more prone to suicidal attempts remains a crucial challenge for prevention and effective intervention^9^. Ribeiro et al.^10^ found that prior self-harm and suicide attempts increase the risk of later attempts, but contribute only marginally to diagnostic accuracy above chance. For this reason, developing tools that can identify signatures of suicide attempt from multimodal data while providing feature-level explanations may represent an important initial step toward clinically useful, validated risk-assessment instruments.

Machine Learning (ML) algorithms for identifying suicide attempters (SAs) versus non-suicide attempters (NAs) have been proposed. A review by Pigoni et al.^11^ showed that most ML models achieved an accuracy of 0.7 or higher. Fortaner-Uyà et al.^12^ developed a model to predict suicide attempts and tested it in a longitudinal study, reaching an accuracy of 0.64 and AUROC of 0.7. However, several methodological issues may affect generalizability of the ML models. One major limitation is overfitting, occurring when a model is excessively tailored to the training dataset, reducing its ability to perform well on unseen data. As an example, the study by Horvath et al.^13^ used a high number of input features (29 features on 353 records), a factor that may have limited the model’s reliability^14^. Another issue is the lack of independent validation, essential to assess a model generalizability beyond the initial training dataset. Many studies fail to incorporate an independent test set, making it difficult to evaluate real-world applicability^15,16^. Additionally, some studies suffer from class imbalance, meaning that the dataset contains significantly more samples from one class than another, which may introduce biases in performance evaluation^17^. For instance, the models developed by Su et al.^18^ and Iorfino et al.^19^ rely on unbalanced datasets, and while some strategies can be used to mitigate this issue, model performance can still be affected. The aforementioned references show that most studies do not adopt rigorous validation approaches, raising questions on the overall applicability of their findings.

Given the lack of a validated BPD-specific tool to identify individuals with a history of suicide attempt, this pilot study tests the hypothesis that a multimodal signature can distinguish lifetime suicide attempters among people with BPD. To explore this idea and its methodological feasibility as a proof-of-concept (POC) for a larger study, we developed DRAMA-BPD (Detecting Retrospective suicide Attempts with Machine learning Approaches in Borderline Personality Disorder), a multimodal, eXplainable Artificial Intelligence (XAI) tool built on a classifier for lifetime suicide attempts among persons with BPD. DRAMA-BPD, trained on the sociodemographic, clinical, and Magnetic Resonance Imaging (MRI) data of 104 individuals with BPD recruited from two cohorts, was designed to overcome most of the limitations previously mentioned, specifically:

- Class imbalance is avoided since our dataset is natively balanced with 47/104 SAs (45%) and 57/104 NAs (55%);
- Overfitting was mitigated by reducing the number of features by means of an extraction process;
- The lack of external validation was not resolved, as we were not able to find an independent compatible dataset. We highlight this as one of the limitations of our study.

While individual techniques are established, their integration represents a methodologically rigorous framework designed to overcome prior limitations and establish feasibility for prospective validation studies. Our approach also aims to evaluate the feasibility of analytic procedures not widely applied in this literature (e.g., MRI harmonization, feature extraction, and ensemble modelling). SHapley Additive exPlanations (SHAP) analysis^20^ was used to interpret feature contributions, making DRAMA-BPD explainable. In light of these considerations, the primary goals of this work are:

- Evaluate the feasibility of deriving an interpretable multimodal signature associated with past suicide attempts;
- Generate preliminary performance estimates to motivate prospective external validation.

## Methods

### Study population

DRAMA-BPD was trained on two cross-sectional samples deriving from previous studies. The CLIMAMITHE study was a multicenter randomized clinical trial (NCT02370316) conducted at two Italian centers, aiming to assess clinical and neurobiological effects of Metacognitive Interpersonal Therapy compared with Structured Clinical Management on 60 individuals with BPD^21,22^. Participants included adults aged 18–45 years who met the Diagnostic and Statistical Manual of Mental Disorders, version IV (DSM-IV)^23^ criteria for BPD, as DSM-5-based version of the instruments were not yet implemented at the time the research protocol was developed. However, the diagnostic criteria for BPD remained virtually unchanged between DSM-IV and DSM-5, ensuring full conceptual and operational comparability^24^. For the purpose of this study, they were divided based on the presence of lifetime suicide attempts recorded in the anamnestic interview and the Structured Clinical Interview for DSM-IV for Personality Disorder (SCID II) (30 SAs and 30 NAs). The CLIMAMITHE study also includes MRI data, consisting of T1-weighted 3D (T13D), Diffusion Tensor Imaging (DTI), and Fluid-Attenuated Inversion Recovery (FLAIR) sequences acquired using a Siemens Skyra 3 T scanner at the Hospital Spedali Civili of Brescia. The CLIMAMITHE protocol was approved by the ethical committee of the coordinating center (Protocol number 67/2014), and informed consent was obtained from all subjects and/or their legal guardian(s).

The SUDMEX_CONN dataset is a case–control study of individuals with cocaine use disorder (ethical number CEI/C/061/2013), and comprises 145 participants who underwent extensive neuropsychiatric assessments^25–27^. Additionally, MRI sequences were acquired, including T13D, 10-min resting-state fMRI, and High Angular Resolution Diffusion Imaging—Diffusion-Weighted Imaging Multishell, acquired with a Philips Ingenia, 3 T scanner at the “National Institute of Psychiatry” in Mexico City. The SCID II scale was used to diagnose BPD. The C9 item from the Mini-International Neuropsychiatric Interview^28^ scale was used to identify SAs. Participants with available MRI data were then selected, resulting in a sample of 44 individuals (17 SAs and 27 NAs). The study was carried out according to the Declaration of Helsinki and was approved by the Ethics Committee of the Instituto Nacional de Psiquiatría “Ramón de la Fuente Muñiz”.

A psychiatrist (AM) and a psychologist (SM), both with extensive experience in the assessment and treatment of individuals with BPD, evaluated the compatibility of the two datasets. We then merged them to increase sample size and statistical power of our exploratory estimates. As the datasets contained different feature sets, only overlapping ones were retained to ensure consistency and minimize biases introduced by missing data imputation. For this reason, only T13D and DTI were selected among MRI features. To mitigate the lack of an independent test set and reduce overfitting risk, we employed well-established methods for performance estimation in small datasets (see *Classifier pipeline* for further details). Merging two populations also introduces heterogeneity in the features that, after harmonization and control for site effects, can improve the generalizability of multimodal signatures.

### Sociodemographic and clinical data

Sociodemographic and clinical data are presented in Table 1. Specifically, the following clinical evaluations were available:

- Difficulties in emotion regulation scale (DERS)^29,30^, a self-administered questionnaire used to assess changes in emotion regulation;
- Barratt impulsiveness scale (BIS)^31^, a 30-item self-administered questionnaire designed to assess impulsivity;
- Symptoms checklist 90 revised (SCL-90-R)^32^, which assesses general psychopathology. This self-administered inventory comprises 90 items across nine symptom dimensions (somatization, obsessive–compulsive tendencies, interpersonal sensitivity, depression, anxiety, hostility, phobic anxiety, paranoid ideation, and psychoticism);
- Structured clinical interview for DSM (SCID), a semi-structured interview to assess major DSM diagnoses. Both datasets used SCID II based on DSM-IV^33^. Specifically, we used all 9 diagnostic criteria for BPD, excluding criterion number 5 (*Recurrent suicidal behavior, gestures, or threats, or self-mutilating behavior*) because of the high correlation with the dependent variable of the classifier.

**Table 1:** Sociodemographic and clinical features of the sample.

Feature	Suicide Attempters (47)	Non-Attempters (57)	p-value
Age [years]	29.43 ± 8.17 (47)	29.77 ± 7.15 (57)	0.676
Sex [% of females]	40% (47)	47% (57)	0.480
Education [years]	11.86 ± 3.07 (43)	12.37 ± 3.14 (49)	0.409
Age at first contact with psychiatric services [years]	21.87 ± 7.13 (45)	24.66 ± 7.96 (50)	0.069
Lifetime acute ward admissions [%]	42% (43)	18% (49)	0.014*
Number of lifetime acute ward admissions	1.28 ± 2.63 (43)	0.65 ± 1.66 (49)	0.027*
Previous psychotherapies [%]	61% (46)	53% (57)	0.404
History of alcohol abuse [%]	63% (46)	46% (57)	0.104
History of substance abuse [%]	66% (47)	68% (57)	0.791
Traumatic and/or stressful experiences [%]	76% (45)	61% (56)	0.116
Self-harm [%]	63% (46)	37% (57)	0.009*
Medical comorbidities [%]	30% (43)	24% (49)	0.539
DERS	106.0 ± 42.3 (47)	100.0 ± 37.5 (57)	0.288
BIS	72.1 ± 11.4 (45)	71.4 ± 12.9 (54)	0.919
SCL-90-R	172.0 ± 71.9 (47)	144.0 ± 68.0 (56)	0.053
BPD criteria from SCID II [% of yes]			
Fear of abandonment	66% (47)	74% (56)	0.394
Unstable relationship	77% (47)	86% (56)	0.237
Unstable Self-Image	89% (47)	86% (56)	0.581
Impulsivity	72% (47)	70% (57)	0.809
Affect instability	87% (47)	86% (57)	0.851
Sense of emptiness	96% (47)	81% (57)	0.022*
Anger	74% (47)	75% (57)	0.901
Transient paranoid ideation/dissociative symptoms	70% (46)	82% (57)	0.126

Sociodemographic and clinical variables of Suicide Attempters and Non-Attempters included in this study. Values in brackets indicate the number of participants for whom the data were available. An asterisk shows features that present a significant p-value (< 0.05). Acronyms: DERS, Difficulties in Emotion Regulation Scale; BIS, Barratt Impulsiveness Scale; SCL-90-R, Symptoms Check-list 90 Revised.

### MRI data

Table 2 reports details about the acquisition equipment for the datasets. MRI scans were processed using standard pipelines to extract structural and connectivity biomarkers, including White Matter hypointensity (WM-hypo) volumes, indicative of brain lesions, from T13D images. Specifically: Freesurfer (v7.3.2) was employed to compute cortical thickness and subcortical volumes based on the Desikan-Killiany atlas^34^, while TRActs Constrained by UnderLying Anatomy (TRACULA, v7.3.2) provided Mean Diffusivity and Fractional Anisotropy measures for major white-matter tracts.

**Table 2:** Acquisition equipments of the two cohorts.

Dataset	Scanner	T13D		DTI	
		Field strength [T]	Voxel size [mm]	Field strength [T]	Voxel size [mm]
CLIMAMITHE	Siemens Skyra	3.0	1 × 1x1	3.0	2 × 2x2
SUDMEX_CONN	Philips Ingenia	3.0	1 × 1x1	3.0	2 × 2x2

MRI sequences available in both CLIMAMITHE and SUDMEX_CONN datasets. Acronyms: T13D, T1-weighted 3D MRI scan; DTI, Diffusion Tensor Imaging.

T1-weighted 3D MRI scans were corrected for smooth intensity variations using *N4BiasFieldCorrection* from Advanced Normalization Tools (ANTs)^35^ and pre-registered to the *MNI_152_T1_1mm* template using the FMRIB Software Library (FSL) *flirt* function with 12 Degrees of Freedom (DOF)^36–39^. Then, *recon-all* function from Freesurfer^36,37^ was used to extract cortical thicknesses and subcortical volumes of regions defined according to the Desikan-Killiany atlas. The *segmentThalamicNuclei* function^40^ extracted thalamic nuclei subunit volumes, and the *segmentHA_T1* function^40,41^ extracted hippocampus and amygdala subfields volumes.

The pre-processing of DTI data involved initial noise removal using the *dwidenoise* function, followed by the elimination of Gibbs artifacts using *mridegibbs* function, both tools from the MRtrix3 package^42^. Subsequently, *dwifslpreproc* (from MRtrix3) function was applied to estimate and correct susceptibility-induced distortions and to remove potential eddy current artifacts. Finally, DTI images were corrected for smooth intensity variations using *dwibiascorrect* function and registered to the *JHU-ICBM-DWI-2 mm* template using flirt with 12 DOF^44^. Because some original DTI images from SUDMEX_CONN exhibited low quality that could affect the registration to the MNI template, we introduced a manual 9 DOF registration of the DTI to the *JHU-ICBM-DWI-2 mm* template between the eddy current removal and the proper flirt registration. TRACULA was then used for reconstructing a set of 42 WM pathways from DTI images, allowing the extraction of Fractional Anisotropy and Mean Diffusivity of WM pathways^43,44^. Image processing pipelines were uniformly applied to both CLIMAMITHE and SUDMEX_CONN datasets, as detailed in Supplemental Information. MRI-derived data underwent quality control (QC) by three expert neuroscientists (AB, SDF, AR) and poor-quality scans or segmentation errors were excluded (2 DTI subject data were discarded). Neuroimaging data were harmonized using the NeuroHarmonize model^45^, with CLIMAMITHE set as the reference due to the elevated prevalence of cocaine use in SUDMEX_CONN (~ 80%). Given that cocaine abuse among BPD individuals typically ranges from 18 to 34%^46,47^, and CLIMAMITHE showed a cocaine abuse prevalence of 25%, was selected as more representative of the general BPD population. Moreover, since this feature differed significantly between the two cohorts (p-value < 0.05), we included the correction of this factor in the harmonization process.

### Classifier pipeline

The combined dataset included 104 individuals (47 SAs, 57 NAs) that passed QC and 345 candidate features. To prevent information leakage, preprocessing, feature extraction, and hyperparameter tuning were carried out exclusively within the training folds of a stratified ten-fold cross-validation, as it offers a favorable balance between bias and variance in performance estimation, and then applied to the corresponding test folds. Preprocessing included encoding of categorical variables, K-Nearest-Neighbour imputation, and min–max scaling to the [0,1] range. Feature extraction, essential to reduce overfitting risk, was performed in two stages. First, we identified the most relevant features using Random Forest (RF) ranking^48^ (number of estimators = 1000, measure used = Mean Decrease in Impurity), keeping the 50 features with the highest score. Then, we removed highly correlated variables using the Variance Inflation Factor (VIF)^49^ (threshold = 5), with an iterative removal, recomputed after each drop. To assess the stability of the process, feature extraction was repeated across folds^50^; the ten features most consistently selected across folds were retained. As our objective was to preserve feature interpretability, we avoided feature extraction methods that combine original variables into latent components, because they prevent direct attribution of model classifications to specific features; one example is Principal Component Analysis (PCA)^51^, which only supports partial interpretability. DRAMA-BPD classifier was implemented by testing several ML models: (1) Support Vector Machine (SVM)^52^, Random Forest, Naive Bayes (NB)^53^, XGBoost^54^, LightGBM^55^, CatBoost^56^, and Regularized Logistic Regression^57^, all tested as individual models; (2) an ensemble of SVM, RF, and NB; and (3) an ensemble of XGBoost, LightGBM, and CatBoost. All these models have been used previously in similar contexts: SVM in^58,59^, RF in^12,17,18^, NB in^58,60^, XGBoost in^61^, LightGBM in^62^, CatBoost in^63^, and regularized logistic regression in^64^. We then selected the best-performing model, meaning the one with highest statistical outcomes, which was the ensemble of SVM, RF, and NB. While these models represent established techniques in psychiatric classification tasks, their integration here is specifically calibrated for BPD-suicide classification with interpretability prioritization. This combination addresses the known gap in BPD-specific ML literature, which has been dominated by single-modality approaches. No covariates were applied in the training process of the models. After training, we applied the SHAP toolkit post-hoc to evaluate features’ relevance and the reciprocal effects in the classification process, with the goal of understanding the model’s decisions and overcoming the current ML black-box approach. SHAP values were computed on held-out test sets and aggregated across splits. Finally, we performed a power analysis by means of Nx Subsampling^65^ to estimate the sample size required to achieve the target accuracy.

### Statistical analysis

Statistical comparisons between variables were performed via the Kruskal–Wallis test, a non-parametric statistical method suitable for cases where the assumptions of normality and homogeneity of variances are not met. The significance level for statistical comparisons was set at 0.05^66,67^. The code used in this study was implemented in Python. Specifically, statistical analyses were conducted with *statsmodels* (0.13.5) and *scipy* (1.7.3), ML models were implemented with *scikit-learn* (1.3.0), and SHAP analysis was performed using the *shap* library (0.41.0).

## Results

### Sociodemographic and clinical features of the sample

Sociodemographic and clinical features of the dataset are shown in Table 1. Statistically significant differences (p < 0.05) emerged for several variables. SAs reported a higher number of lifetime acute ward admissions, were more likely to have experienced at least one acute psychiatric hospitalization, engaged more frequently in self-harming behaviors, and more commonly endorsed a sense of emptiness, a core BPD criterion.

### Classifier pipeline

We initially aimed to reduce the number of features to ten, but results showed that using fewer features led to similar performance. Therefore, we opted for a total of four features, for the sake of simplicity. The most relevant features were related to MRI and clinical data, while sociodemographic and DTI-derived features did not contribute to the model:

- Thickness of the right hemisphere rostral anterior cingulate (RH_rACC)
- Volume of the left hemisphere presubiculum (LH_PRS)
- Volume of white matter hypointensity (WM-hypo)
- Symptoms checklist 90 revised (SCL-90-R)

Table 3 shows data related to the relevant features of the two groups.

**Table 3:** Relevant features of the combined sample.

Feature	Suicide attempters (47)	Non-attempters (57)	p-value
RH_rACC thickness [mm]	2.84 ± 0.23 (46)	2.70 ± 0.17 (57)	< 0.001*
LH_PRS volume [mm^3^]	221 ± 32 (46)	238 ± 29 (57)	0.013*
WM-hypo volume [mm^3^]	1630 ± 440 (46)	1480 ± 392 (54)	0.081
SCL-90-R	172 ± 72 (29)	144 ± 68 (30)	0.053

Averages and standard deviations of the most relevant features of Suicide Attempters and Non-Attempters involved in this study. Values in brackets indicate the number of participants for whom the data were available. An asterisk shows features that present a significant p-value (< 0.05). Acronyms: RH_rACC, Right Hemisphere Rostral Anterior Cingulate; LH_PRS, Left Hemisphere Presubiculum; SCL-90-R, Symptoms Check-list 90; WM-hypo, White Matter hypointensities*.*

The best-performing ensemble classifier yielded the following results over ten-fold cross-validation: accuracy = 0.67 (95% CI 0.63—0.71), sensitivity = 0.58 (95% CI 0.53—0.63), specificity = 0.77 (95% CI 0.67—0.86), and Area Under the ROC Curve 0.68 (95% CI 0.63—0.72). The balanced accuracy is 0.68 (95% CI 0.63—0.72). Performance metrics for each class are as follows:

- Class 1 (SA): sensitivity = 0.58 (95% CI 0.53–0.63), specificity = 0.77 (95% CI 0.67–0.86), PPV = 0.69 (95% CI 0.59–0.81), NPV = 0.67 (95% CI 0.62–0.74)
- Class 0 (NA): sensitivity = 0.77 (95% CI 0.67–0.86), specificity = 0.58 (95% CI 0.53–0.63), PPV = 0.67 (95% CI 0.62–0.74), NPV = 0.69 (95% CI 0.59–0.81)

**Fig. 1:**
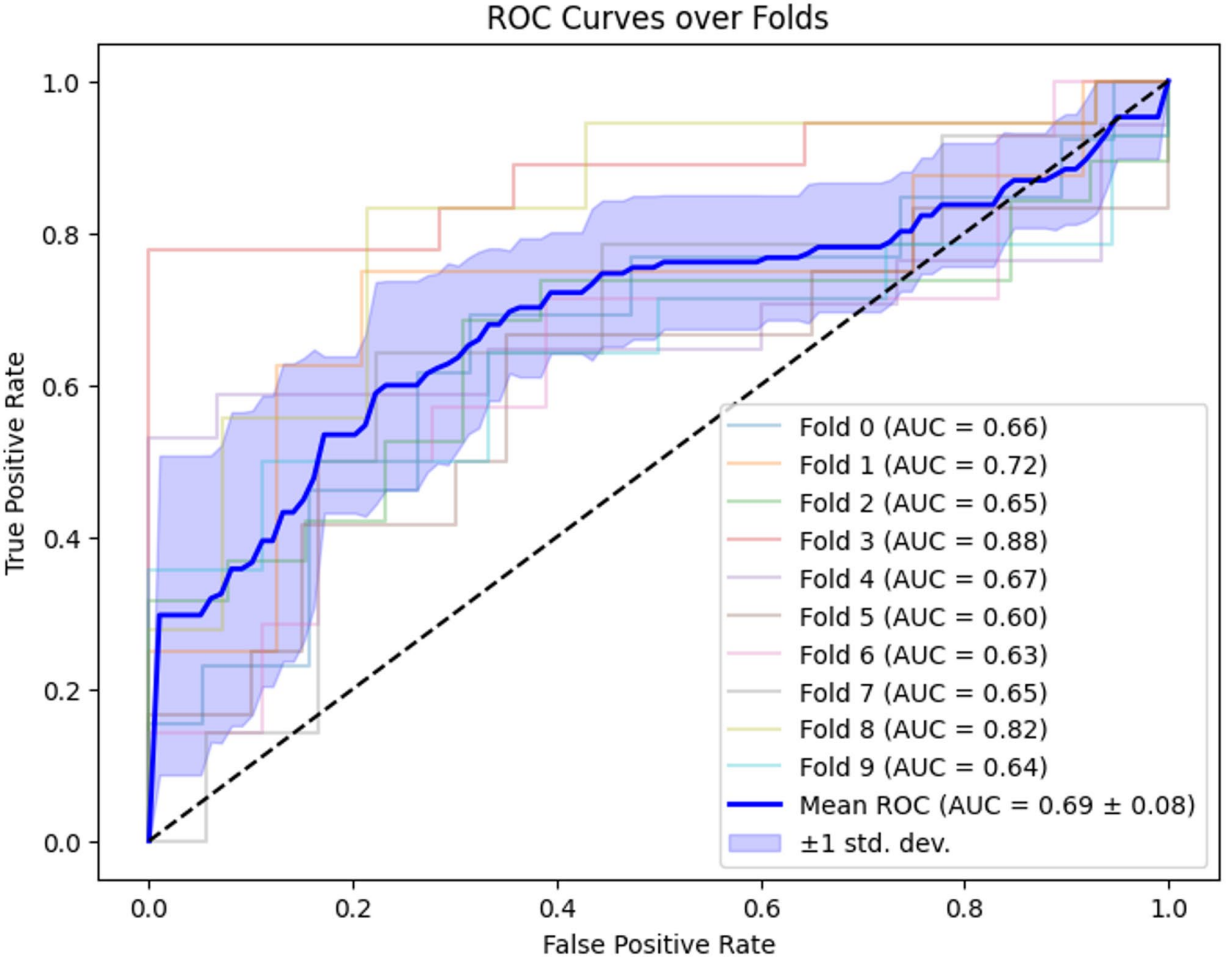
ROC Curves over folds. The ROC curve for every fold is reported with different colors, and the corresponding AUC value in brackets. The blue curve represents the mean ROC, with the light blue area representing the standard deviation.

Figure 1 shows the ROC curves of every fold and reports the average one. Power analysis indicated that the target accuracy of 0.85, in line with reliable clinical tools^68^, would be reached with a dataset 8 times larger. Further details can be found in Supplemental Information.

### Feature relevance

**Fig. 2:**
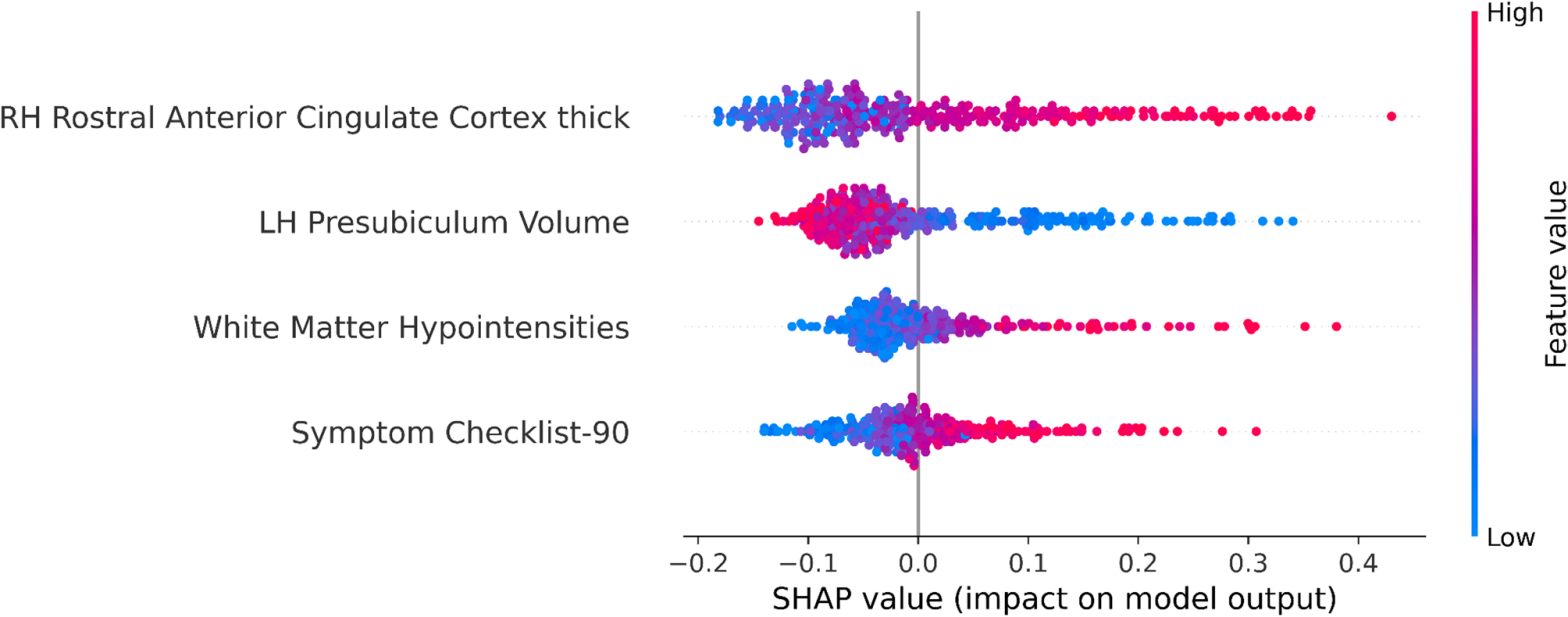
Contribution of each feature to the classification, represented by the SHAP summary plot. Acronyms: RH, Right Hemisphere; LH, Left Hemisphere.

We analyzed the SHAP summary plot to evaluate feature importance (Fig. 2). Features are ranked by relevance, with RH_rACC thickness identified as the most influential, followed by LH_PRS volume, WM-hypo volume, and SCL-90-R. Each point represents an individual’s feature value, where color denotes feature values (red: high, blue: low) and the x-axis indicates impact on model output. High RH_rACC thickness values cluster on the right, indicating strong association with SA classification. A similar pattern was observed for SCL-90-R and WM-hypo volume, both suggesting increased SA likelihood. In contrast, higher LH_PRS volumes were associated with NA classification.

**Fig. 3:**
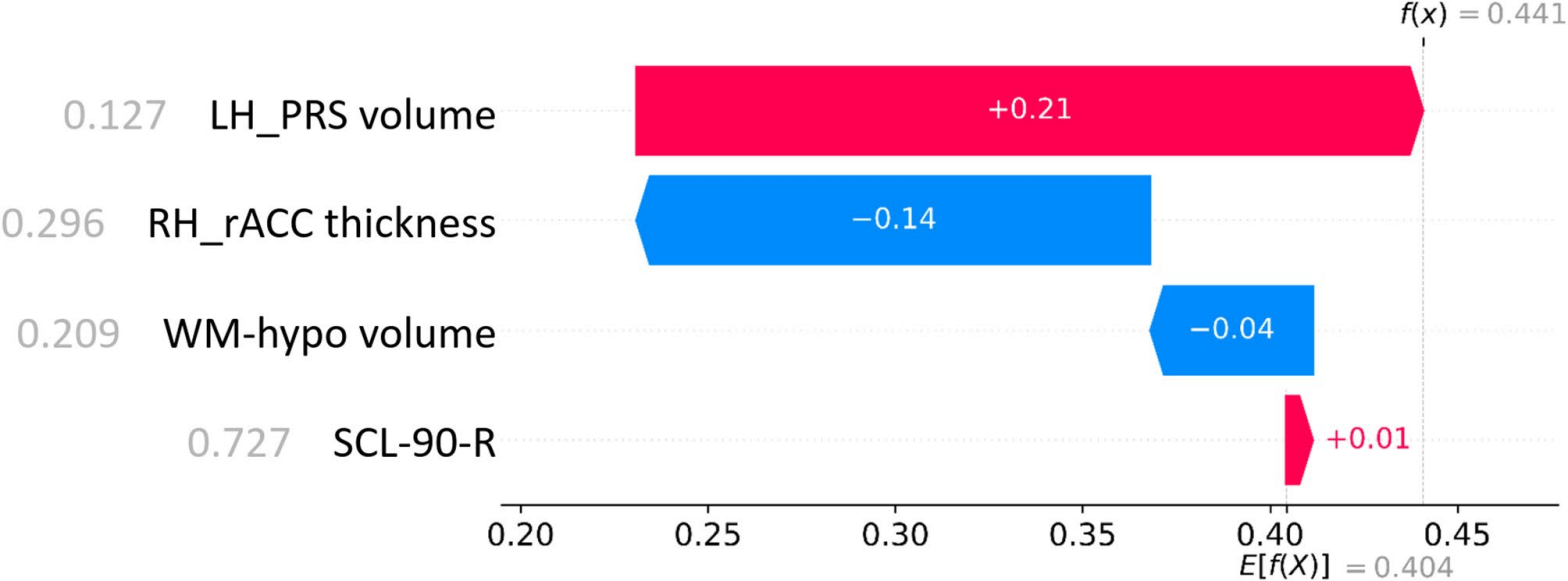
Contribution of each feature to the correct classification of a NA subject (True Negative). The numbers left to the features names are their scaled values, while values on the red/blue bars are the SHAP weights. Acronyms: RH_rACC, Right Hemisphere Rostral Anterior Cingulate; LH_PRS, Left Hemisphere Presubiculum; WM-hypo, White Matter hypointensities; SCL-90-R, Symptoms Check-list 90; NA: Non-Attempter.

**Fig. 4:**
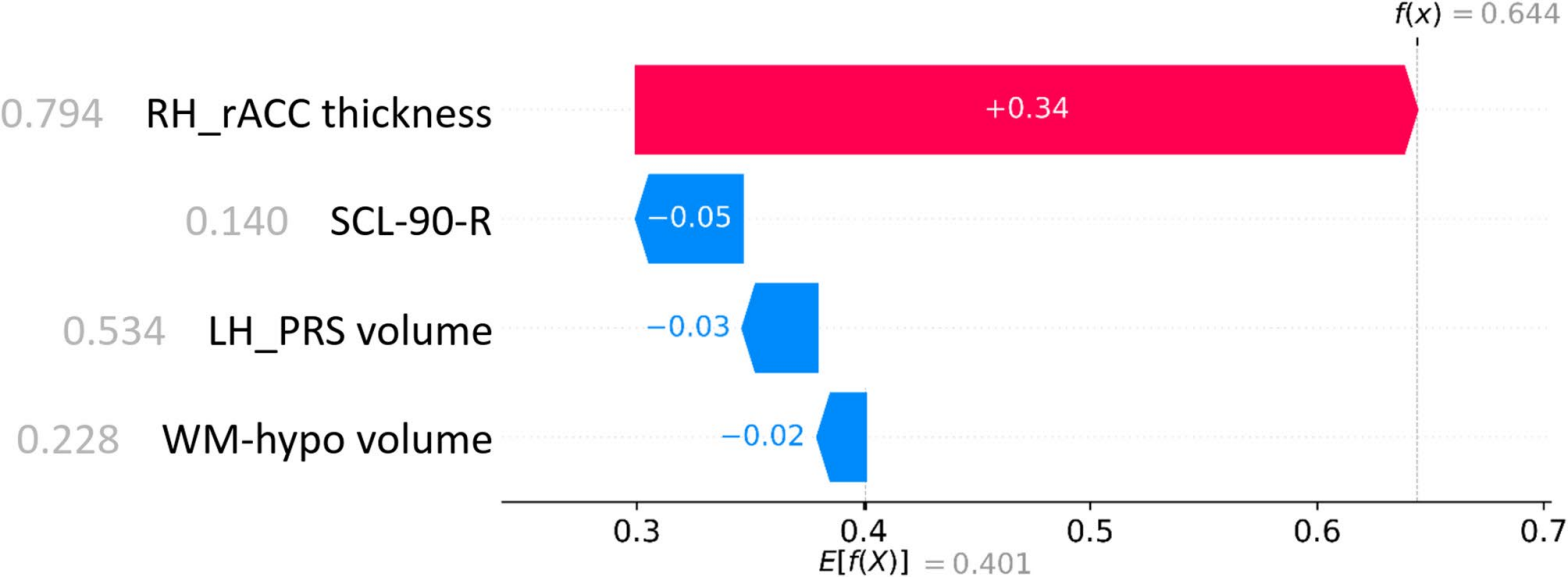
Contribution of each feature to the correct classification of a SA subject (True Positive). The numbers left to the features names are their scaled values, while values on the red/blue bars are the SHAP weights. Acronyms: RH_rACC, Right Hemisphere Rostral Anterior Cingulate; LH_PRS, Left Hemisphere Presubiculum; WM-hypo, White Matter hypointensities; SCL-90-R, Symptoms Check-list 90; SA, Suicide Attempter.

Figure 3 shows the scaled feature values and corresponding SHAP contributions for a True Negative (TN) classification, i.e. a NA correctly classified as a NA. DRAMA-BPD base expectation for the output is 0.404, while low LH_PRS volume (scaled value = 0.127, on a range from 0 to 1) has the strongest positive contribution (+ 0.21), pushing the classification towards the SA class. Conversely, low RH_rACC thickness (scaled value = 0.296) contributes negatively (-0.14), pushing the classification towards the NA class. WM-hypo volume (scaled value = 0.209) and SCL-90-R (scaled value = 0.296) show smaller contributions (− 0.04 and + 0.01, respectively). The combined effect of these features results in a final value of 0.441, resulting in a correct NA classification. Figure 4 presents a similar analysis for a True Positive (TP) classification, i.e. a SA correctly classified as a SA. In this case, high RH_rACC thickness (scaled value = 0.794) strongly supports SA classification with a positive contribution (+ 0.34). SCL-90-R (scaled value = 0.140), LH_PRS volume (scaled value = 0.534), and WM-hypo volume (scaled value = 0.228) push the classification towards NA class with small contributions (-0.05, -0.03, and -0.02, respectively). The combined effect of these features results in a final value of 0.644, meaning a correct SA classification. These examples illustrate how individual features influence classification outcomes and highlight potential biases in the model’s decision-making process.

## Discussion

The goal of this study was to demonstrate that a multimodal approach, combined with robust data processing and ML techniques, can classify lifetime suicide attempters in people with BPD. To do this, we used sociodemographic, clinical, and MRI data from the CLIMAMITHE and SUDMEX_CONN studies to train the DRAMA-BPD model, an ensemble classifier of lifetime suicide attempters among people with BPD. Feature selection was applied to reduce the number of features and avoid overfit. The process was data-driven, and thus the importance of each feature was calculated by the RF model (see Methods for further details). Interestingly, the present study showed that neuroimaging has a key role in this classification. Indeed, among the 4 most influential variables, 3 are related to neuroimaging. DTI-derived features did not contribute to the model. This could be because DTI-derived features are sensitive to acquisition parameters^69–72^ and motion artifacts^73^, causing a limited classification contribution in this specific setup. SHAP was applied post-hoc to help interpret the results of the classifier.

### Right hemisphere rostral anterior cingulate cortex

DRAMA-BPD’s explainability analysis using SHAP identified Right Hemisphere Rostral Anterior Cingulate Cortex (RH_rACC) thickness as the top contributor to model output. The Rostral Anterior Cingulate Cortex (rACC) is a medial frontal subregion implicated in emotion regulation, cognitive control, and decision-making^74^. Literature suggests a potential link between rACC structural alterations and suicidality in BPD. Soloff et al.^75^ reported reduced rACC Gray Matter (GM) in suicidal BPD individuals versus Healthy Controls (HCs), but found no rACC difference between BPD SAs and NAs, whereas an increased volume has been associated with aggression in high-lethality attempters^76^. Duarte et al.^77^ reported an association between increased rACC and suicide attempts in BD type 1 patients, suggesting a potential compensatory mechanism. In DRAMA-BPD, higher rACC thickness produced positive SHAP attributions, thereby increasing the probability estimate for SA class. While this result converges with reports linking rACC morphology to suicidal behaviour, findings remain inconsistent across studies, and further research is required to clarify these discrepancies. Possible explanations for increased GM volumes or thickness are neuroinflammatory processes, inefficient synaptic pruning mechanisms during neurodevelopment, or neuroplasticity-driven adaptations in response to early-life adversity, as discussed in^78^. Authors suggest that hyperactivity of ACC and insula may lead to changes in brain plasticity and hypertrophy as compensatory mechanisms^79–81^. It should be noted that studies^77^ and^78^ focused on volumetric analysis of the whole rACC, while in our case the feature extraction process identified rACC thickness, limiting the direct comparability of the two findings.

### Left hemisphere presubiculum

The presubiculum, a subregion of the parahippocampus, is implicated in memory formation^82^. Several studies have documented hippocampal volume alterations in BPD. Brambilla et al.^83^ reported reduced hippocampal volumes, particularly in BPD subjects with childhood abuse, while Rossi et al.^84^ localized significant differences mainly to the CA1 sector (with differences reaching up to 10–20%). These studies used different field strengths (1.5 T) and segmentation protocols than ours (3 T), limiting direct comparison. Bøen et al.^85^ (3 T) analyzed and identified significant volume reductions in the dentate gyrus and cornu ammonis (CA), though the all-female sample used may limit generalizability. In a similar sample, O’Neill et al.^86^ observed reductions in the left hippocampal head and body, as well as the bilateral hippocampal tail. Ruocco et al.^87^ conducted a meta-analysis of 11 MRI studies, comprising 205 individuals with BPD and 222 HCs, revealing an average 11% reduction in hippocampal volume, a difference that was unaffected by psychotropic medication use. Our data revealed a significant difference in Left Hemisphere Presubiculum (LH_PRS) volume, with SAs showing lower average volumes than NAs. SHAP attributions show that lower LH_PRS volumes increase the probability that DRAMA-BPD classifies an individual as an SA. In summary, there is strong evidence to support smaller hippocampal volume as a marker for BPD, although studies investigating its direct relationship with suicidal behavior are lacking.

### White-Matter hypointensities

Hypointensities in T1-weighted images, corresponding to hyperintensities in T2-weighted or FLAIR sequences, typically represent WM lesions. These can include various pathologic features or anatomic structures, such as calcifications, hemorrhages, gliosis, and scar tissues^88^, which are common findings in aging and dementia, but evidence of higher WM hypointensities in BPD compared to controls is lacking. This may denote incidental findings without clinical relevance, explaining why this variable contributes marginally to the model. Nevertheless, Grangeon et al.^89^ conducted a meta-analysis of MRI studies, and concluded that individuals with deep WM hyperintensities and periventricular hyperintensities have a higher association with suicidal behaviors, even though the underlying mechanisms remain unclear. Pompili et al.^90^ also showed that periventricular hyperintensities are strongly associated with suicide attempts. In our sample, larger volumes of WM-hypointensities were observed in SAs. SHAP attributions indicate that larger WM-hypo volume modestly increased the DRAMA-BPD probability of SA. In light of this, our findings are partially consistent with literature, since WM-hypointensities are associated with suicide attempts. However, a study that investigates this aspect specifically in the BPD population is lacking. This emphasizes the need for deeper investigation and analysis of how WM-hypo volume influences classification, and the SHAP-based association identified here should be considered hypothesis-generating.

### Symptoms Check-list 90 Revised

The SCL-90-R is not specifically designed to assess BPD symptomatology and does not include items specific to suicide. However, it captures general trait-psychopathology across multiple domains, several of which are relevant to suicidal behavior^32^. These include depression, focusing on symptoms generally associated with an increased suicide risk (hopelessness, sadness, feelings of worthlessness), hostility and aggression (potentially linked to suicide in individuals with impulsivity or emotional regulation difficulties), anxiety, and psychoticism. In this study, we used the total score of the SCL-90-R scale as a feature for our dataset. Nevertheless, to compare our results with literature, we analyzed the subscales for statistical differences. Consistently with Lee et al.^91^ (Korean SCL-90-R^92^), we observed higher hostility and paranoia scores among SAs, although these differences did not reach statistical significance in our sample. SCL-90-R contributed modestly to DRAMA-BPD classifications according to SHAP, with higher subscale scores pushing the model toward a SA classification.

### Summary and result interpretation

DRAMA-BPD shows moderate predictive performance, with a balanced accuracy of 0.68 (95% CI 0.63–0.72) and AUC of 0.68 (95% CI 0.63–0.72). While the confidence intervals for sensitivity (0.58, 95% CI 0.53–0.63) and other metrics indicate some overlap and uncertainty, particularly in identifying TPs, the model shows a clear signal above chance, especially for specificity (0.77, 95% CI 0.67–0.86). The moderate predictive performance could be mainly explained by the intrinsic complexity of predicting suicide attempts in BPD and limited sample size. Nevertheless, these results suggest that the classifier captures meaningful patterns in the data, proving that a multimodal signature could identify suicide attempters. These results are preliminary: the sample size is modest (N = 104), cohorts were cross-sectional and merged across sites, and no external prospective validation is available. Therefore, we frame our findings as hypothesis-generating and evidence that may support future works. Interestingly, the feature extraction process identified one clinical feature, while the remaining were all MRI-derived. This represents a potential change of perspective with regards to existing literature, which mostly relied on sociodemographic and clinical data only. However, this pattern should be interpreted cautiously and validated in independent datasets. ML model explainability can support the interpretability and potential clinical usefulness. This may be especially relevant in complex conditions such as BPD, where medical decisions may be influenced by both biological and behavioral data. By using SHAP, we identified the influence of single features on DRAMA-BPD, allowing us to move beyond black-box predictions to a more interpretable approach. This factor is especially important in clinical practice, where understanding a model’s prediction may help the clinician’s decision^93,94^. However, SHAP-based interpretability does not substitute for neurobiological investigations, which requires independent validation and targeted multimodal studies. For this reason, this evidence provides an initial foundation for XAI in BPD, though larger and more diverse datasets are needed to support clinical use.

### Limitations

Overall, the robustness of the pipeline and validation strategy we employed reinforces the relevance of the identified brain regions in BPD, but some limitations must be addressed. The first is the small size of the analyzed dataset, although comparable with similar works from literature. This is caused by several factors, most notably the difficulties in recruiting people with BPD and the cost of MRI 3 T procedures. Regarding the CLIMAMITHE dataset, the outpatient nature and long duration of the interventions required a high level of adherence and commitment, which implies a certain rate of refusals and dropouts. In addition to that, the comprehensive clinical and neuroimaging assessments required by the study protocol were time-consuming and demanding, which further reduced the pool of eligible and willing participants. Our experience of slower recruitment aligns with previous findings in the BPD literature. Woo et al.^95^ report that stigma, external referral pathways, and the high procedural burden are key barriers to recruitment and retention in BPD research. Regarding MRI post-hoc harmonization tools, we acknowledge that our sample size is relatively small for multisite neuroimaging^96^, potentially limiting the power of harmonization to fully eliminate site-specific variance, and the lack of external validation prevents us from determining whether identified features generalize to new sites or scanners. Moreover, power analysis showed that to reach a target accuracy of 0.85, in line with reliable clinical tools, the dataset size would need to be enlarged to 800 individuals with BPD (balanced between SAs and NAs). This suggests that future work should prioritize expanding the sample size and identifying and validating more informative biomarkers, potentially represented by Affect-Modulated Startle^97^. From a ML perspective, the lack of validation on an independent dataset may limit the generalizability of the model. Future studies should incorporate comparisons with external, compatible datasets to assess the broader applicability of DRAMA-BPD. Regarding XAI analysis, because SHAP describes associations learned by the model rather than causation, models including these features appear to achieve higher accuracy, suggesting that they may capture patterns relevant to the classification. Nonetheless, these results should be treated as hypothesis-generating; replication in independent, prospective cohorts and targeted multimodal investigations are required before inferring biological mechanisms or clinical utility. An additional limitation is the lack of longitudinal validation for DRAMA-BPD. Indeed, our goal was not to train a suicide predictor; rather, it was to identify a multimodal signature that could potentially pave the way for developing tools that help clinicians forecast future suicidal behaviors. A longitudinal study could fill this gap, transforming the present classifier model to an actual predictor. Finally, future research could examine the interactions among the four identified features, optimize decision thresholds to reduce their influence on classification outcomes, and explore advanced modeling or feature-engineering strategies. We emphasize that this is a pilot exploratory analysis, thus our results primarily inform feasibility and estimates for future prospective validation studies rather than immediate clinical deployment.

## Conclusions

Suicide is one of the leading causes of death among individuals with BPD, yet accurately identifying those at risk remains a major challenge. Existing ML approaches primarily focus on other psychiatric disorders, and often suffer from methodological limitations, including overfitting and limited model interpretability. In this pilot study, we developed DRAMA-BPD, an ensemble classifier combining SVM, RF, and NB, trained on a cross-sectional, multimodal dataset, formed by merging CLIMAMITHE and SUDMEX_CONN. To prioritize interpretability, we adopted a feature extraction strategy, with findings consistent with previous studies. SHAP was used to investigate feature contributions, providing an initial foundation for XAI in BPD lifetime suicide attempts classification. Because of the modest sample size, cross-sectional design, and absence of independent prospective validation, we frame these results as hypothesis-generating. DRAMA-BPD illustrates that multimodal, interpretable ML can identify patterns associated with a lifetime history of suicide attempt in BPD, but further work is needed to improve overall performance through larger samples and enriched clinical predictors, and to validate findings in external and prospective cohorts. Until external validation, DRAMA-BPD should be considered an exploratory tool that may help prioritize future research rather than a ready-to-use clinical instrument.

## Supplementary Information

Below is the link to the electronic supplementary material.

Supplementary Material 1

## Data Availability

The SUDMEX_CONN dataset is publicly available from OpenNeuro at https://openneuro.org/datasets/ds003037/versions/1.0.0. Access and reuse are subject to the terms and conditions posted on the OpenNeuro repository. The CLIMAMITHE dataset used in this study is available upon request through the NewPsy4U platform at https://newpsy4u.eu. Following registration, users may submit an access request via the platform interface, which will be reviewed and approved at the discretion of the PI, Roberta Rossi, last author of this paper.
