# Supplementary material for: An explainable multimodal artificial intelligence model for classifying suicide attempters with borderline personality disorder: a pilot study
Source: Sci Rep. 2025 Dec 19;16:1902. doi: 10.1038/s41598-025-31550-9 (PMC12804717; doi:10.1038/s41598-025-31550-9)
Supplement: Supplementary file 1 — Supplementary Material 1 [file 41598_2025_31550_MOESM1_ESM.docx]

**An Explainable Multimodal Artificial Intelligence Model for Classifying Suicide Attempters with Borderline Personality Disorder: a Pilot Study**

**Supplementary Materials**

**Quality Control:**

Quality control of the outputs was performed retrospectively, inspecting the results of each pipeline slice by slice and discarding those of low quality or incorrect segmentation (2 DTI subject data were discarded).


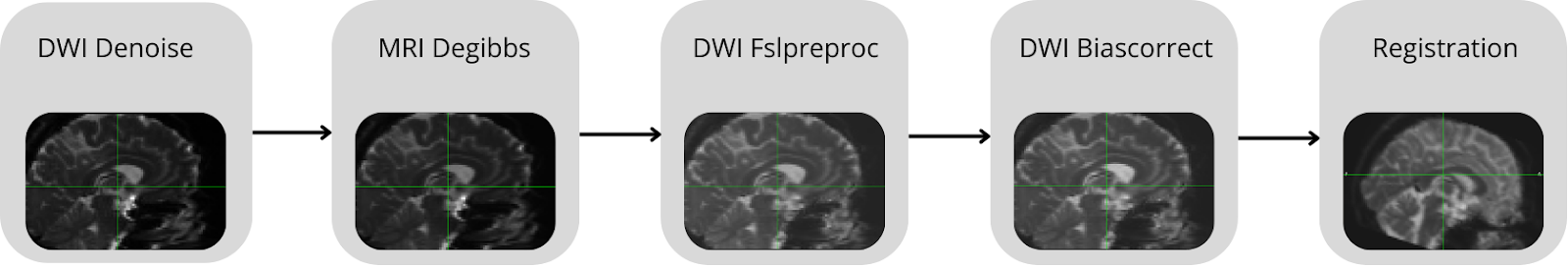


*Supplementary figure 1 - DTI preprocessing steps*

**Power analysis**

The power curve has been calculated by means of Nx Subsampling [1]. We used three points of the training set (75% of the merged dataset): 25%, 50% and 100%, corresponding to 19, 39, and 78 records, respectively. To prevent overfit, the number of features has been reduced accordingly; 1, 3, and 4 features, respectively, from most to least relevant. Results for accuracy are the following:

- 25% of the training set: 0.591 ± 0.051
- 50% of the training set: 0.634 ± 0.070
- 100% of the training set: 0.697 ± 0.079

The power curve is shown in Supplementary figure 2. The results show that the original dataset size should be increased by 8 times to reach a target accuracy of 0.85, in line with the best performing works in literature, resulting in a total of approximately 800 BPD patients.


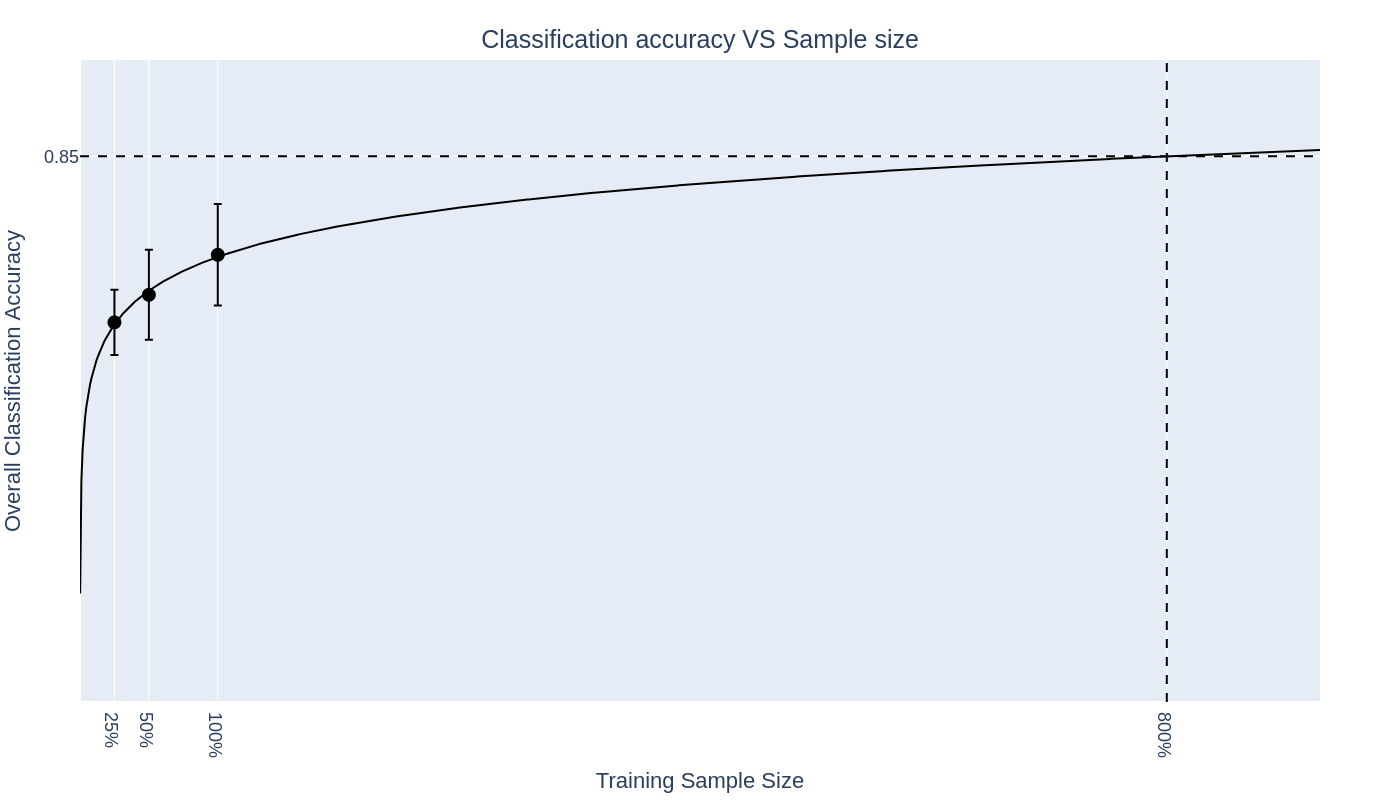


*Supplementary figure 2 - Power curve of our dataset showing the intercept with 0.85 accuracy.*

**Supplementary references**

[13] Balki I, Amirabadi A, Levman J, Martel AL, Emersic Z, Meden B, et al (2019): Sample-size determination methodologies for machine learning in medical imaging research: A systematic review. Can Assoc Radiol J 70(4): 344-353.
